# Supplementary figures and images for: The proprotein convertase BLI-4 promotes collagen secretion prior to assembly of the Caenorhabditis elegans cuticle
Source: PLoS Genet. 2023 Sep 18;19(9):e1010944. doi: 10.1371/journal.pgen.1010944 (PMC10538796; doi:10.1371/journal.pgen.1010944)

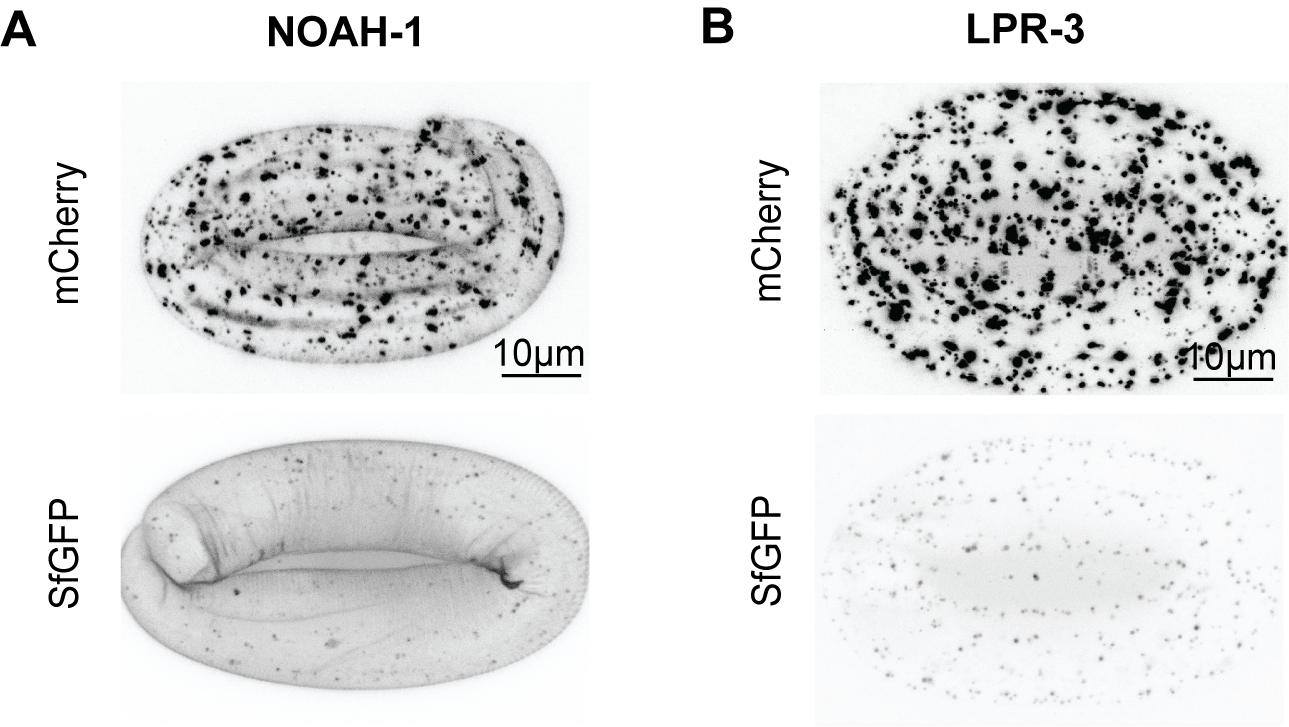

Supplement: S1 Fig — A,B) Pre-cuticle mCherry fusions, but not SfGFP fusions, mark large internal structures in late embryos (4 hours after 1.5-fold). A) noah-1(mc68 [NOAH-1::mCherry(int)]) compared to aaaIs25 [NOAH-1::SfGFP(int)]. B) lpr-3(cs266 [ss::mCherry::LPR-3]) compared to lpr-3(cs250 [ss::SfGFP::LPR-3]). All images are maximum intensity projections from confocal Z-slices and representative of at least 5 embryos per genotype. Scale bar, 10 microns. (TIF) [file pgen.1010944.s003.tif]

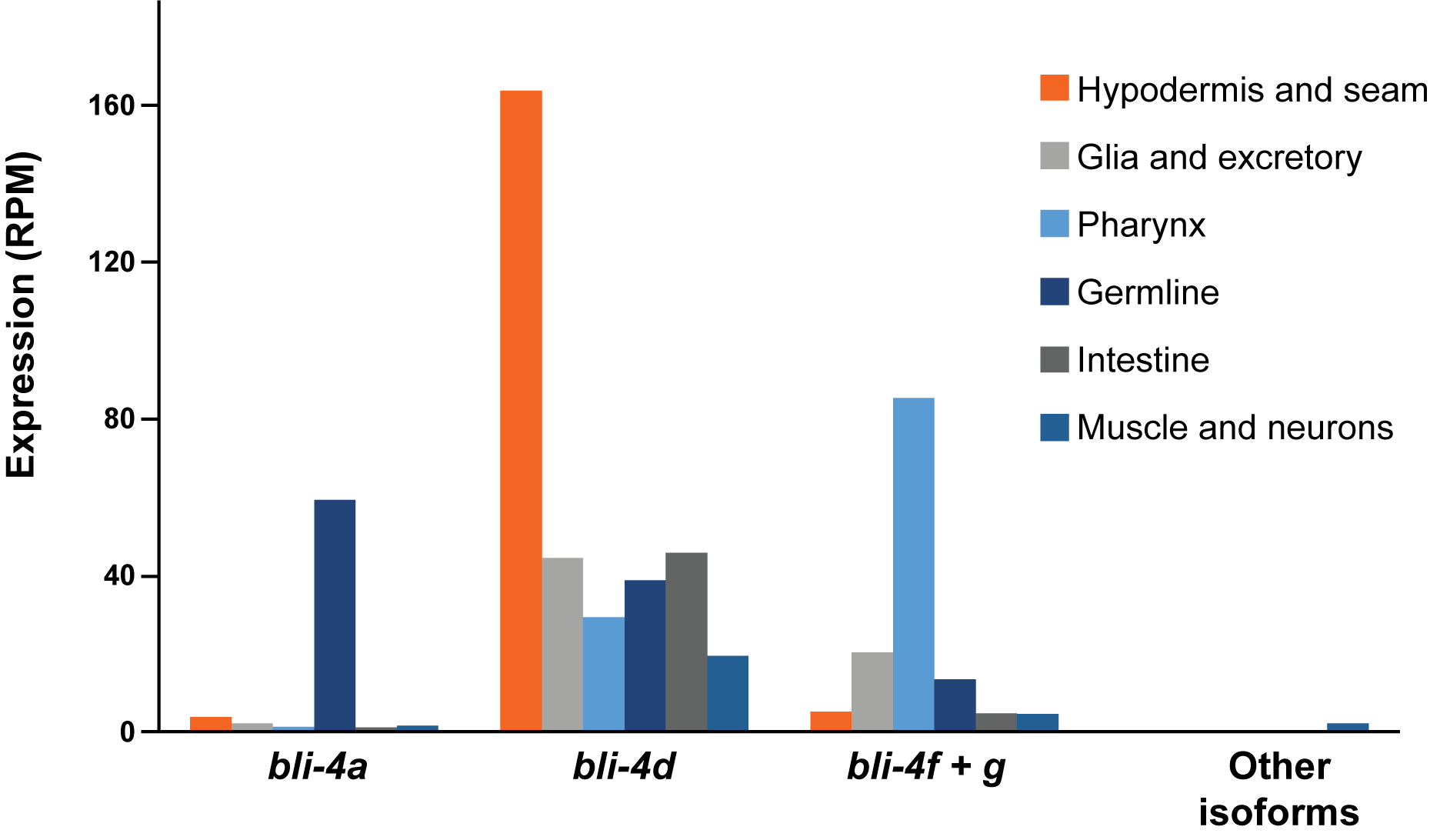

Supplement: S2 Fig — Embryo scRNA-seq reads from [52] were mapped to the 3’ ends of the various bli-4 isoforms to estimate expression levels in the major tissue classes (Methods). RPM, reads per million. Our methods could not distinguish isoforms f and g, despite their different 3’ coding regions, since these isoforms share a similar 3’ UTR. The most highly expressed isoform in the embryo and in the epidermis was the CRD-containing isoform, bli-4d, with more modest levels of bli-4a and f/g, and negligible evidence for other isoforms. Note that comparing absolute levels of one isoform with another should be done with caution due to transcript specific biases, and the scRNA-seq data comprise exclusively cells from the first ~half of embryogenesis, so later differences in expression will not be captured. (TIF) [file pgen.1010944.s004.tif]

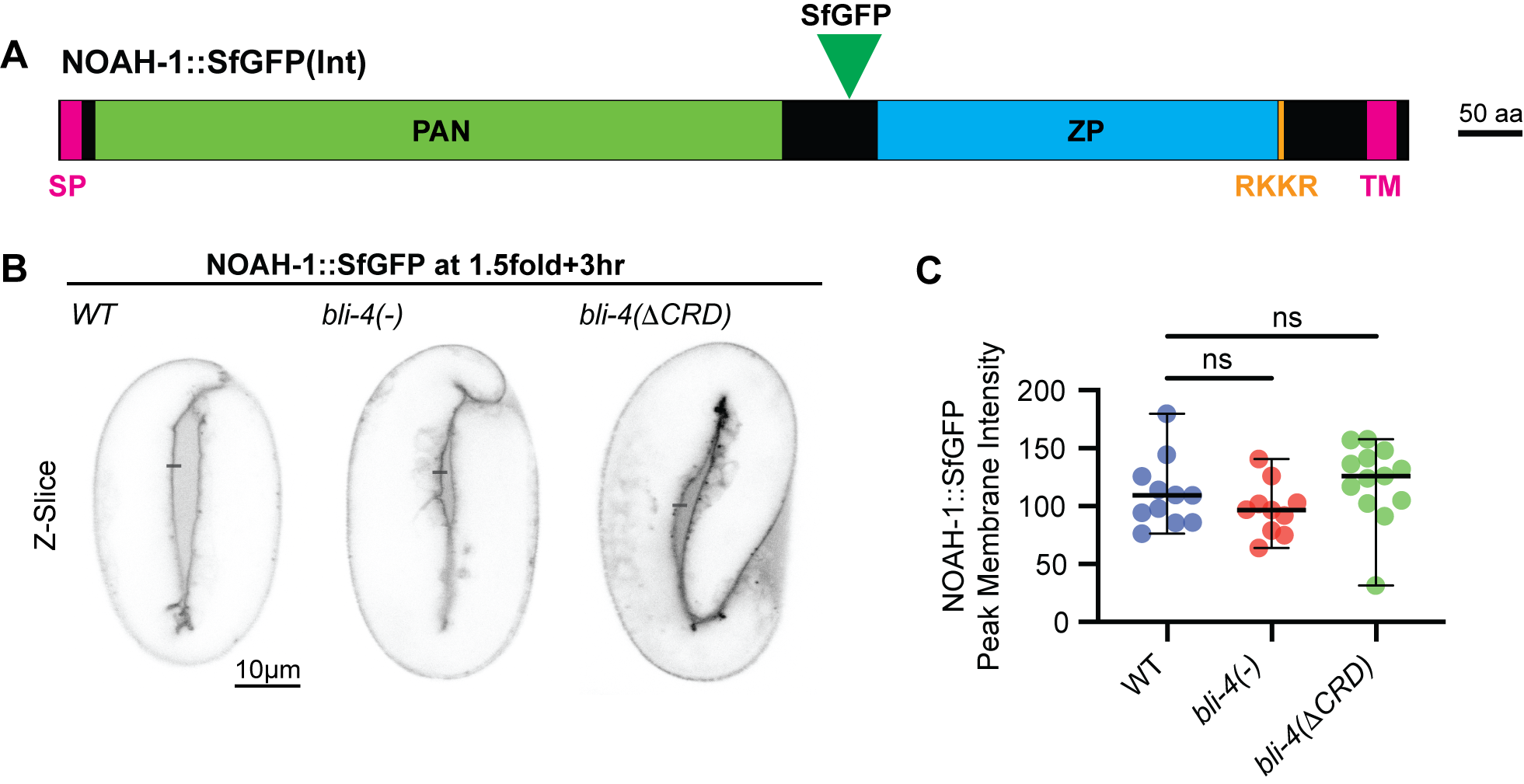

Supplement: S3 Fig — A) Schematic diagram of the NOAH-1::SfGFP(int) transgene fusion (aaaIs25) [87], which was used instead of the endogenous fusion because of chromosomal linkage of the bli-4 and noah-1 loci. The SfGFP tag (green triangle) is located between the Plasminogen (PAN) and ZP domains, as indicated. The C-terminal CFCS (RKKR, orange) is located before a predicted transmembrane (TM) domain. B) NOAH-1::SfGFP(int) appears similar between WT and bli-4 mutant embryos (1.5-fold + 3 hour stage). Images are single confocal Z-slices and are representative of at least 10 embryos per genotype. C) Peak membrane fluorescence intensity was calculated with FIJI [84] using line scans across single confocal Z-slices, as shown in B. There was no significant difference (ns) among the genotypes. (TIF) [file pgen.1010944.s005.tif]
